# Tramadol for Refractory Restless Legs Syndrome Following Methadone Intolerance: A Case Report

**DOI:** 10.7759/cureus.114310

**Published:** 2026-08-10

**Authors:** Binh Pham, Vivian Thai

**Affiliations:** 1 Sleep Medicine, Southwest Healthcare Medical Education Consortium, Temecula, USA

**Keywords:** carbidopa and levodopa, chronic insomnia, comorbid anxiety, gabapentin, methadone, migraine disorder, pregabalin, restless legs syndrome (rls), ropinirole, tramadol

## Abstract

Restless legs syndrome (RLS) refractory to guideline-directed therapy may require treatment with low-dose opioids. Methadone is an established option for refractory RLS but may be limited by medication intolerance. We present a 52-year-old woman with refractory RLS who failed multiple pharmacologic therapies, including ropinirole, carbidopa/levodopa, gabapentin, and pregabalin. Methadone produced excellent symptom control but was discontinued because of dysphoria, emotional lability, cognitive slowing, and persistent daytime grogginess. Transition to tramadol 50 mg nightly resulted in sustained symptom control without recurrence of these adverse effects, allowing discontinuation of gabapentin and improved daytime functioning. This case highlights the importance of individualized opioid selection in refractory RLS and suggests that tramadol may be an effective alternative for carefully selected patients with methadone intolerance.

## Introduction

Restless legs syndrome (RLS), also known as Willis-Ekbom disease, is a chronic neurologic sensorimotor disorder characterized by an irresistible urge to move the legs that worsens during periods of rest, improves with movement, and is typically more pronounced in the evening or at night [1,2]. Affecting approximately 5% to 10% of adults, RLS is a common cause of sleep disturbance and impaired daytime functioning. Diagnosis is established using standardized clinical criteria after exclusion of potential mimics and secondary causes [2].

Current management emphasizes identification and correction of iron deficiency when present, followed by pharmacologic therapy tailored to symptom severity and frequency. Recent American Academy of Sleep Medicine guidelines recommend α2δ calcium channel ligands as first-line therapy for chronic persistent RLS, whereas dopaminergic agents are used more selectively because of the risk of augmentation during long-term treatment. For patients with refractory RLS who experience persistent symptoms despite guideline-directed therapy or who are unable to tolerate standard treatments, low-dose opioid therapy may be considered under careful clinical supervision [3-5].

Methadone has demonstrated efficacy in patients with refractory RLS and is an accepted therapeutic option when conventional therapies fail [6]. However, long-term treatment success depends not only on symptom control but also on medication tolerability, as adverse effects may ultimately limit continued use. Reports describing successful transition from methadone to tramadol because of methadone intolerance remain limited. We present a case of refractory RLS that failed multiple guideline-supported therapies and achieved sustained symptom control with tramadol following methadone discontinuation because of treatment-limiting neuropsychiatric adverse effects.

## Case presentation

A 52-year-old woman with a history of anxiety and migraines presented for evaluation of an eight-year history of RLS. She described an irresistible urge to move her legs that occurred predominantly during the evening and overnight, frequently awakening her between 2:00 and 3:00 AM. She initially described the sensation as feeling *fidgety* and later as *ants crawling* in her legs without associated pain. Stretching her calves, forcefully flexing her legs, and walking around the house provided temporary relief. Although she had little difficulty initiating sleep, nocturnal RLS symptoms caused nightly sleep-maintenance insomnia of variable severity. She denied upper-extremity involvement, impulsive behaviors, parasomnias, dream enactment, and excessive daytime sleepiness.

Her habitual sleep schedule was approximately midnight to 8:30 AM, with three nocturnal awakenings related to RLS symptoms or nocturia. She did not nap during the day. She denied snoring, witnessed apneas, nocturnal choking or gasping, and morning dry mouth. Given the absence of clinical features suggestive of sleep-disordered breathing, sleep apnea testing was not pursued. Her Epworth Sleepiness Scale (ESS) score was 4/24 [7]. She consumed several cups of coffee each morning but avoided caffeine after 10:30 AM. She denied tobacco and alcohol use.

On initial evaluation, she was 5 ft 1 in (155 cm) tall, weighed 98 lb (44.5 kg), and had a body mass index of 18.5 kg/m². Her blood pressure was 114/78 mmHg, pulse was 70 beats/minute, and oxygen saturation was 99% on room air. Physical examination demonstrated a healthy-appearing woman in no acute distress. Cardiopulmonary examination was unremarkable, and neurologic examination was normal.

Before presentation, the patient had undergone multiple medication trials with either inadequate efficacy or poor tolerability, as summarized in Table 1. At the initial evaluation, she was able to recall the names and general clinical effects of her prior RLS treatments but could not reliably recall the exact chronology, duration, or dosage of several earlier medication trials. Accordingly, historical treatment details are reported to the extent available from the clinical history. Over-the-counter RLS preparations and magnesium supplementation were ineffective. Ropinirole failed to improve her symptoms. Carbidopa/levodopa initially produced symptomatic benefit; however, its efficacy diminished over time with recurrence of nocturnal symptoms. She denied earlier symptom onset during the day, spread of symptoms to previously unaffected body regions, or impulse-control behaviors while receiving dopaminergic therapy. Gabapentin provided partial improvement at doses up to 1,600 mg daily but caused excessive grogginess. Pregabalin 75 mg daily, administered in combination with gabapentin and carbidopa/levodopa, failed to provide adequate symptom control. At presentation, her medication regimen consisted of gabapentin 1,200 mg nightly, pregabalin 75 mg daily, and carbidopa/levodopa 50/200 mg nightly. Because of persistent symptoms despite multiple pharmacologic therapies, carbidopa/levodopa was tapered with plans for discontinuation, and methadone 2.5 mg nightly was initiated.

**Table 1:** Summary of pharmacologic therapies administered throughout the patient's clinical course for restless legs syndrome (RLS). Methadone provided excellent symptom control but was discontinued because of persistent neuropsychiatric adverse effects despite dose adjustment. Tramadol subsequently achieved sustained symptom control without recurrence of these adverse effects at the most recent clinical and telephone follow-up.

Medication	Dose	Clinical response	Reason for discontinuation/current status
Over-the-counter RLS preparations	Not available	Ineffective	Persistent symptoms
Magnesium supplementation	Not available	Ineffective	Persistent symptoms
Ropinirole	Not available	No clinical improvement	Ineffective
Carbidopa/levodopa	50/200 mg nightly	Initial symptom improvement followed by loss of efficacy	Persistent symptoms
Gabapentin	Up to 1,600 mg nightly	Partial symptom improvement	Excessive daytime grogginess
Pregabalin	75 mg daily	Inadequate symptom control	Persistent symptoms
Methadone	2.5 mg nightly	Excellent control of nocturnal RLS symptoms	Dysphoria, emotional lability, daytime grogginess, cognitive slowing, reduced motivation
Tramadol	50 mg nightly	Sustained symptom control without reported adverse effects	Not applicable; treatment ongoing

At follow-up, methadone produced substantial improvement in nocturnal RLS symptoms. However, the patient developed significant daytime grogginess, cognitive slowing, reduced motivation, dysphoria, and emotional lability despite adequate symptom control. During a brief postoperative period following foot surgery, methadone was temporarily withheld while she received hydrocodone/acetaminophen for postoperative pain. During this period, she reported resolution of the daytime cognitive symptoms, which recurred after methadone was resumed. To improve tolerability, methadone tablets were transitioned to an oral solution to permit dose reduction to approximately 1 to 1.5 mg administered earlier in the evening. Low-dose gabapentin was also prescribed as needed for breakthrough symptoms.

Despite these interventions, the patient ultimately discontinued methadone because of persistent dysphoria, emotional lability, daytime grogginess, cognitive slowing, and loss of interest in previously enjoyable activities. Following methadone discontinuation, daytime alertness improved; however, nocturnal RLS symptoms recurred despite treatment with gabapentin 800 mg at 7:00 PM and 500 mg at 10:00 PM. She also reported intermittent use of sequential compression devices, which provided partial symptomatic relief.

The patient verbally reported that recent iron studies obtained by her neurologist demonstrated a serum iron level of 75 µg/dL, total iron-binding capacity of 300 µg/dL, and a ferritin level of 89 ng/mL. The original laboratory report, institution-specific reference ranges, and additional laboratory data were unavailable for independent review. Tramadol 50 mg nightly was subsequently initiated.

At her most recent follow-up, the patient reported complete control of nocturnal RLS symptoms with tramadol 50 mg nightly without recurrence of dysphoria, daytime grogginess, or emotional lability. She successfully discontinued gabapentin and reported improved daytime alertness and overall functioning. A subsequent telephone follow-up confirmed sustained symptom control and continued good medication tolerability.

## Discussion

Successful management of refractory RLS depends not only on achieving adequate symptom control but also on maintaining long-term medication tolerability. In this patient, methadone provided excellent control of nocturnal RLS symptoms but was ultimately discontinued because of persistent neuropsychiatric adverse effects despite multiple dose-adjustment strategies. Transition to tramadol resulted in sustained symptom control with improved daytime functioning and without recurrence of these adverse effects. Although conclusions cannot be generalized from a single case, this experience suggests that individualized opioid selection may be considered when methadone is clinically effective but poorly tolerated.

Current treatment guidelines recommend correction of iron deficiency when present, followed by pharmacologic therapy based on symptom severity and frequency. α2δ calcium channel ligands are recommended as first-line therapy for chronic persistent RLS, whereas dopaminergic agents are used more selectively because of the risk of augmentation during long-term treatment. For patients with refractory RLS who continue to experience clinically significant symptoms despite guideline-directed therapy or who are unable to tolerate standard treatments, carefully monitored low-dose opioid therapy is an accepted treatment option [3-5]. The clinical course of our patient closely reflects this recommended stepwise treatment approach, progressing through multiple evidence-based therapies before escalation to opioid treatment.

Methadone is an established treatment for refractory RLS after failure of conventional therapies. Ondo reported sustained symptomatic improvement with low-dose methadone in patients with refractory RLS, supporting its role as an effective rescue therapy [6]. Long-term follow-up by Silver et al. similarly demonstrated durable clinical benefit with methadone and dopamine agonists over 10 years in appropriately selected patients [8]. More recently, data from the National RLS Opioid Registry demonstrated sustained efficacy and relative dose stability of low-dose opioids in patients with refractory RLS receiving long-term treatment [9]. Collectively, these studies support methadone as an effective long-term treatment option for appropriately selected patients with refractory RLS. Our patient's experience was consistent with these findings, as methadone effectively controlled nocturnal symptoms; however, persistent daytime grogginess, cognitive slowing, dysphoria, emotional lability, and reduced motivation ultimately outweighed its therapeutic benefit despite dose reduction, conversion to an oral solution, and earlier evening administration.

Evidence supporting tramadol for the treatment of RLS remains considerably more limited than that supporting methadone and other opioids. In an open-label study, Lauerma and Markkula reported clinical improvement in patients with RLS treated with tramadol, suggesting that it may represent a therapeutic option in selected patients [10]. Unlike conventional opioids, tramadol exerts weak μ-opioid receptor agonism and inhibits serotonin and norepinephrine reuptake [11]. A positron emission tomography study in nonhuman primates demonstrated central serotonin and norepinephrine transporter occupancy following tramadol exposure, further supporting its mixed opioid and monoaminergic pharmacology [12]. Although the precise mechanism by which tramadol improves RLS remains uncertain, this distinct pharmacologic profile may partly explain the favorable clinical response observed in our patient. Consistent with this pharmacologic profile, transition to tramadol resulted in sustained symptom control without recurrence of the neuropsychiatric adverse effects experienced with methadone, allowing successful discontinuation of gabapentin and improvement in daytime alertness.

Although tramadol was well tolerated in our patient, clinicians should recognize that augmentation has been reported during long-term tramadol therapy [13,14]. Continued clinical follow-up remains important to identify earlier symptom onset, increasing symptom severity, or spread of symptoms to previously unaffected body regions regardless of the specific opioid selected for refractory RLS.

This report has several limitations. As a single-patient case report, the findings cannot establish comparative efficacy between methadone and tramadol. Symptom severity was assessed clinically without use of a standardized instrument such as the International Restless Legs Syndrome Rating Scale (IRLS) [15]. Additionally, the exact chronology, duration, and dosage of several historical medication trials could not be reliably established from the patient's retrospective recall. The patient verbally reported recent iron studies obtained through her neurologist, but the original laboratory report was unavailable for independent review. Consequently, institution-specific reference ranges and additional laboratory parameters could not be independently verified. Nevertheless, the patient underwent multiple medication trials, longitudinal follow-up across several clinical encounters, and subsequent telephone confirmation of sustained benefit, providing a detailed description of individualized opioid management in refractory RLS.

## Conclusions

This case demonstrates successful management of refractory RLS with tramadol following methadone intolerance. Although methadone provided excellent symptom control, persistent neuropsychiatric adverse effects limited its long-term use despite dose optimization. Transition to tramadol resulted in sustained symptom control, improved daytime functioning, and successful discontinuation of gabapentin. While larger studies are needed to better define the role of tramadol in refractory RLS, this case highlights the importance of individualized opioid selection based on both therapeutic efficacy and medication tolerability.
